# Social Media Efficacy in Crisis Management: Effectiveness of Non-pharmaceutical Interventions to Manage COVID-19 Challenges

**DOI:** 10.3389/fpsyt.2021.626134

**Published:** 2022-02-07

**Authors:** Yunye Zhou, Anca Draghici, Jaffar Abbas, Riaqa Mubeen, Maria Elena Boatca, Mohammad Asif Salam

**Affiliations:** ^1^Law School, Chongqing University, Chongqing, China; ^2^Faculty of Management in Production and Transportation, Politehnica University of Timisoara, Timisoara, Romania; ^3^School of Media and Communication, Shanghai Jiao Tong University, Shanghai, China; ^4^School of Management, Harbin Institute of Technology, Harbin, China; ^5^Faculty of Economics and Administration, King Abdulaziz University, Jeddah, Saudi Arabia

**Keywords:** COVID-19, crisis management, social media use, non-pharmaceutical interventions, health related information

## Abstract

The new identified virus COVID-19 has become one of the most contagious diseases in human history. The ongoing coronavirus has created severe threats to global mental health, which have resulted in crisis management challenges and international concerns related to health issues. As of September 9, 2021, there were over 223.4 million patients with COVID-19, including 4.6 million deaths and over 200 million recovered patients reported worldwide, which has made the COVID-19 outbreak one of the deadliest pandemics in human history. The aggressive public health implementations endorsed various precautionary safety and preventive strategies to suppress and minimize COVID-19 disease transmission. The second, third, and fourth waves of COVID-19 continue to pose global challenges to crisis management, as its evolution and implications are still unfolding. This study posits that examining the strategic ripostes and pandemic experiences sheds light on combatting this global emergency. This study recommends two model strategies that help reduce the adverse effects of the pandemic on the immune systems of the general population. This present paper recommends NPI interventions (non-pharmaceutical intervention) to combine various measures, such as the suppression strategy (lockdown and restrictions) and mitigation model to decrease the burden on health systems. The current COVID-19 health crisis has influenced all vital economic sectors and developed crisis management problems. The global supply of vaccines is still not sufficient to manage this global health emergency. In this crisis, NPIs are helpful to manage the spillover impacts of the pandemic. It articulates the prominence of resilience and economic and strategic agility to resume economic activities and resolve healthcare issues. This study primarily focuses on the role of social media to tackle challenges and crises posed by COVID-19 on economies, business activities, healthcare burdens, and government support for societies to resume businesses, and implications for global economic and healthcare provision disruptions. This study suggests that intervention strategies can control the rapid spread of COVID-19 with hands-on crisis management measures, and the healthcare system will resume normal conditions quickly. Global economies will revitalize scientific contributions and collaborations, including social science and business industries, through government support.

## Introduction

The World Health Organization, China office reported a new strain of SARS infection (severe acute respiratory syndrome) in Wuhan, China, in late December 2019 (1–5). Accordingly, health experts recognized this fatal viral disease as SARS-CoV-2 (6–8). This new strain of the viral disease caused COVID-19, which brought the ongoing worldwide pandemic (9–11). The WHO declared the outbreak as an “International Concern” related to the Public Health Emergency on January 30, 2020. WHO later called it a global pandemic on March 3, 2020 (12–15). The rapid spread of COVID-19 has massively infected people worldwide and caused public anxiety similar to the 1918 H1N1 influenza plague instigated by an “H1N1 Influenza A” viral disease (16–18). The Spanish influenza pandemic persisted from February 1918 to April 1920. This viral disease infected 500 million people. In four successive waves, the H1N1 pandemic infected about one-third (33%) of the worldwide population. The death toll is typically reported to be somewhere between 20 and 100 million and made it the deadliest pandemic in recorded human history (19). Data released by the WHO and John Hopkins University showed that there were over 223,474,638 verified COVID-19 cases, a total of 4,611,342 deaths, and more than 200,010,731 recovered patients as of September 9, 2021, worldwide (20). The advent of the COVID-19 lethal communicable viral disease has resulted in the most dangerous and fatal human health disaster in the current century of human history (21).

Not since World War II has the world faced more significant global health difficulties and economic crisis management, including social, political, environmental, and financial and health emergency issues. This infectious viral disease has massively interrupted social interactions, business activities, global relations, and global economies (22–25). The main feature of COVID-19, similar to that of MERS and SARS, was its rapid spread worldwide through air travel. The 1918 Spanish influenza pandemic took months longer to spread in Europe, Australia, and South America because ship-borne travel took a long time. However, in the modern world, fast air travel permits voyagers to traverse this globe within a day or less. The key reason for the rapid spread of COVID-19 across continents was that screening efforts at airports were relatively costly and unsuccessful (26). The common feature of in-flight transmission of the COVID-19 disease among passengers was also evident during the SARS outbreak (27, 28).

## Symptoms of COVID-19 and Recovery Ratio

The symptoms of the ongoing lethal disease COVID-19 are not static and vary from mild to moderate to severe infection (4). The typical virus infection symptoms include muscular pain, congestion, runny nose, body aches, nausea or vomiting, diarrhea, headache, loss of taste and smell, nasal congestion, cough, rhinorrhea, fever, sore throat, and breathing complications. The general symptoms can be different among sufferers, and their symptoms might change with time. For people without prior throat and nose disorders, loss of smell combined with the loss of taste indicates an association with the COVID-19 infection with a specificity of 95% (29). The literature specified that almost 81% of people present with mild to moderate COVID-19 infection symptoms, including mild pneumonia, and 14% display severe disease symptoms, such as hypoxia, dyspnea, or 50% lung involvement imaging. A minority of the people (5%) face very acute symptoms, including shock, respiratory failures, or multi-organ dysfunction (30). About 33% of COVID-19-infected people do not show observable virus symptoms at any point (31).

Therefore, the asymptomatic carriers of COVID-19 tend not to undergo testing and therefore spread the infectious disease to others (32). Infected people will show symptoms of the infection later, called “pre-symptomatic,” or develop mild symptoms and spread the virus (33). It is a common phenomenon of virus infections that an infected person will show symptoms later. The median delay time is 4–5 days for COVID-19 symptoms. After exposure to the virus, most symptomatic patients experience symptoms within the first to the third week. Almost all infected people will experience one sign of the virus within the first 12 days (34, 35). More than 90% of infected people recover from the acute infection phase, depending on their immune system. However, some patients experience the adverse effects of COVID-19 infection for several months after they recover due to damage to organs. Hence, multi-year investigations are underway to further explore the long-term adverse impacts of the COVID-19 disease worldwide (35).

## Global Mental Health Challenges Amid the COVID-19 Outbreak

The arrival of COVID-19 posed social, environmental, financial, and mental health challenges worldwide (36). In the first wave, there were more than 119.7 million declared infected people, with a death toll of more than 2.7 million worldwide. However, almost 97% of patients survived the infection of this lethal disease which indicated that 96.3 million people recovered as of March 12, 2021. The second and third waves became more dangerous and lethal. As of July 18, there were over 191,084,631 cases, more than 4,103,306 deaths, and 174,041,354 recovered cases worldwide (14). COVID-19 has dramatically changed people's health-related behavior and lifestyle (37). Educational institutions, such as schools and universities, have been closed and exams and activities have been postponed (38). Health information services have been limited, socializing with friends and a more comprehensive range of mixing with friends and families has been discouraged. Living in these situations can be difficult for young people's social, physical, and mental health (39). This study analyzed information and resources related to COVID-19 that can support people through these challenging times and inspire them to become leaders in addressing the uncertainties of the COVID-19 pandemic.

Past research identified the relationship between MERS (Middle East respiratory syndrome) and SARS to cause psychiatric and mental health problems (40). The severe MERS and SARS phases resulted in psychiatric symptoms, such as mental distress, depression, mood disorder, confusion, anxiety, psychotic symptoms, panic attacks, stress, and delirium (41–44). A study identified post-illness problems, such as fatigue, PTSD (post-traumatic stress disorder), memory impairment, anxiety, depression, and irritability (43). The COVID-19 outbreak resulted in neurological manifestations, dysomia, parageusia, encephalitis, cerebrovascular disease, and actuate meningitis. It has led to mental health issues worldwide (45, 46). A past study examined the COVID-19-enhanced psychiatric complications in the UK. The altered mental status among people was the second most prevalent problem. It caused brain disorders, such as encephalitis and encephalopathy. Over 50% of the patients facing altered mental status were over 60 years (47). The psychological responses during past infection diseases can offer helpful insights into mental health research (6, 48). The Ebola virus illness epidemic outbreak during 2014–2016 hampered public health efforts to manage fear-related human behaviors and impeded survivors' recovery. Past follow-up research has reported persistent mental health problems among infectious diseases survivors, and over 50% of Ebola disease patients reported various mental disorders, such as depression, anxiety disorder, and post-traumatic stress disorder (49). The survivors of SARS reported levels of mental health problems, such as anxiety disorders, depression, panic attacks, social isolation, irritation, and post-traumatic symptoms (8, 50). In follow-up studies over 8 years, almost 25% of SARS survivors reported mental health problems, such as post-traumatic stress disorder (51, 52).

Evidence from past pandemics and infectious disease arrivals has reported adverse mental health consequences due to social isolation and other restrictions in human society (53–55). Large-scale disease outbreaks, pandemics, and natural and traumatic crises cause higher levels of mental health issues, such as depression, anxiety, domestic violence, child abuse, stress, and substance use disorder (56). Amid the COVID-19 outbreak, physical distancing, restriction on economic activities and social gatherings, and closure of educational institutions increased the prevalence of anxiety, stress, PTSD, antisocial and irritating behaviors, depression, and suicidal thoughts among all age groups (57–59). A nationwide psychological disorders survey based on the Chinese population amid the COVID-19 pandemic identified a series of mental health issues, including depression, anxiety disorders, antisocial behaviors, panic disorders, and PTSD (60). The past research findings indicated an almost 200% increase in depression and anxiety disorders among adults during the quarantine restrictions (61). The COVID-19 outbreak in the UK evidenced higher levels of mental health issue symptoms among the adult population in 2020 (62). The mental health problems' prevalence related to the pandemic indicated the level of mental disorders in the general population. The United States of America, Census Bureau Household Pulse Survey reported a spike in anxiety disorders, antisocial behaviors, depression, and stress in April 2020. Almost 35% of US residents reported the clinical symptoms of depression, mental distress, and anxiety disorders during January, February, and March 2019. The fatal result of the COVID-19 virus has affected the general population's mental health (63, 64). Therapeutic mental health care and timely preventive measures are helpful to address the general population's mental health needs in the COVID-19 outbreak (65). The prompt response to control the adverse effects of the COVID-19 crisis on mental health, psychological education, and timely preventive measures can help decrease infection risks and promote resilience to the influence of social changes necessitated by the COVID-19 outbreak (66).

**Figure 1:**
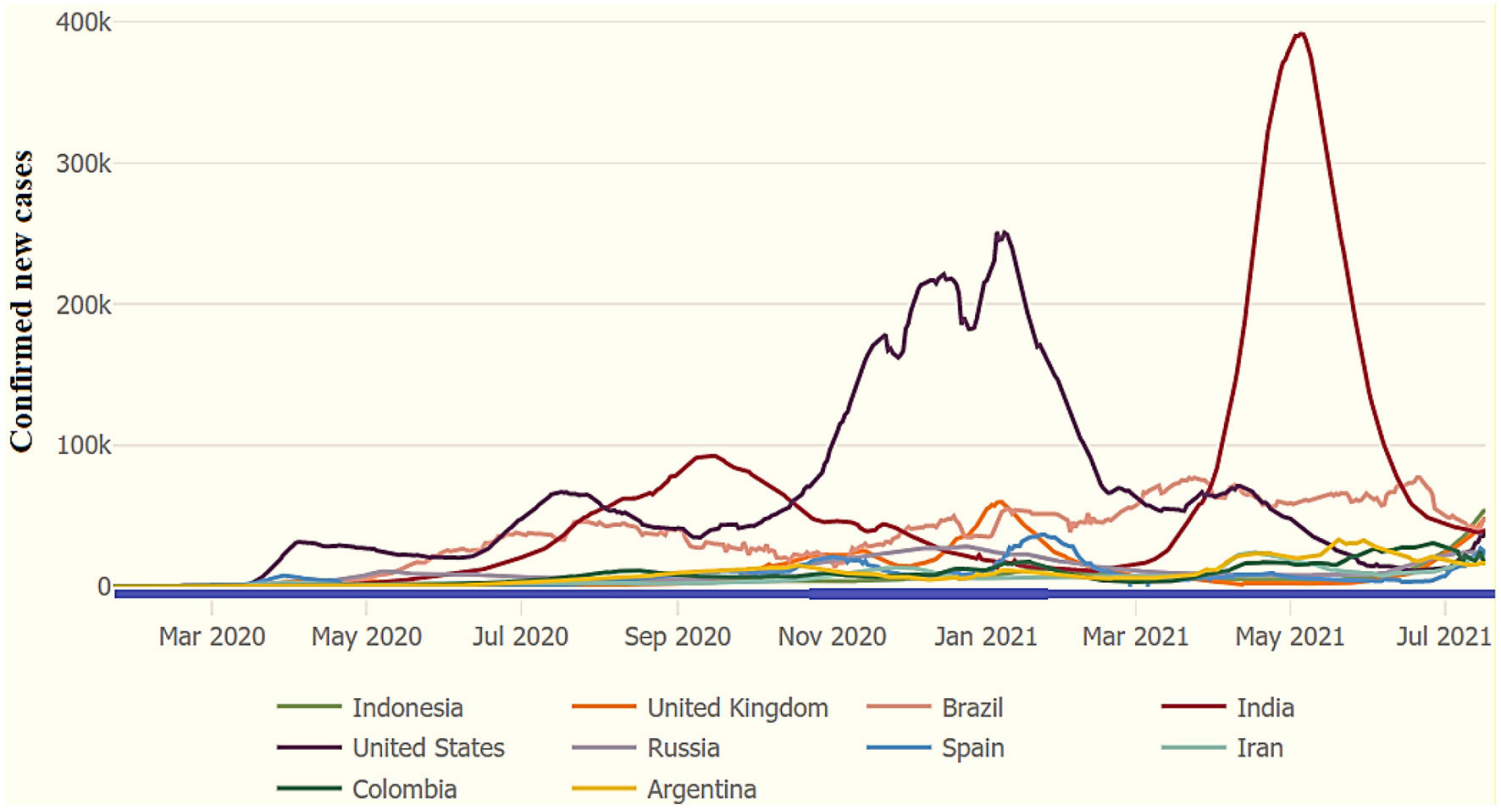
COVID-19: Top 10 most affected countries (7-day moving average), as of July 18, 2021. Source: WHO and JHU COVID-19 datasets.

Figure 1 shows the 10 most affected countries around the world. The US is still the most affected with confirmed COVID-19 patients as of January 31, 2021. The second most affected is India, as indicated in Figure 1. Followed by the UK, Italy, Spain, France, and Brazil. Globally, countries have initiated plans to “flatten the COVID-19 pandemic cases curve.” Flattening the COVID-19 curve involves minimizing the transmission and reducing new cases of COVID-19 from 1 day to the next day. Reducing new cases of infectious disease helps prevent healthcare systems from becoming overwhelmed. When fewer COVID-19 positive patients emerge today than were reported on the previous day, it is a sign that the state/country is flattening the curve. A flattened curve shows a downward trend of recording new positive cases of the coronavirus. The 7-day moving average analysis visualizes recent COVID-19 cases, and this method reflects the rate of change. This research helps manage healthcare systems to provide medical treatment to the patients, and it is helpful to lower the burden on healthcare systems around the world.

WHO facts and figures about COVID-19 stipulate that the United States has the highest number of confirmed coronavirus patients. It reported 29,300,000 patients, with 532,400 deaths and a case mortality rate of 1.80%, the highest number of any nation and the 10th highest in terms of per capita income. Recently, India has reported the most significant number of infected patients in Asia. India has the second largest number of coronavirus patients in the world. There have been 11,308,846 total testified cases, 158,306 deaths, and a case mortality rate of 1.40%. Brazil has the third highest COVID-19 case rate globally and declared 11,277,717 confirmed cases, with a death toll of 272,889 and a case mortality ratio of 2.405. Health experts first identified a patient with the coronavirus in Russia on January 31, 2020. Since then, Russia has confirmed 4,311,893 COVID-19-positive patients, with a death toll of 89,224 and a CFR (case fatality ratio) of 2.10%. Similarly, the United Kingdom has reported 4,254,714 confirmed coronavirus cases, with 125,403 deaths and a corresponding mortality rate of 2.90%, as of March 12, 2021. As of July 18, there were over 191.1 million verified coronavirus cases, more than 4.1 million deaths, and 174.0 million recovered patients.

Table 1 shows the COVID-19 positive cases, deaths, and mortality rate by considering the top 20 affected countries, as of July 18, 2021. According to JHU and the WHO's statistics, the USA remains the highest affected country and reported 34,067,912 positive COVID-19 cases, with 608,884 total deaths and a case-fatality ratio (CFR) of 1.80%. India is the second most affected country with 31,106,065 declared cases, 413,609 deaths, and a CFR of 1.30%. Brazil remains the third highest affected country with 19,342,448 positive cases, 541,266 deaths, and a CFR of 2.80% as of July 18, 2021. See Figure 1 for more details.

**Table 1:** COVID-19 verified cases and rate of mortality in the top 20 affected countries (July 18, 2021).

**Country**	**Confirmed**	**Deaths**	**Case-fatality**	**Deaths/100k**
	**cases**		**(%)**	**pop**.
United States	34,067,912	608,884	1.80	185.5
India	31,106,065	413,609	1.30	30.27
Brazil	19,342,448	541,266	2.80	256.46
France	5,917,397	111,657	1.90	166.5
Russia	5,860,113	145,222	2.50	100.59
Turkey	5,522,039	50,488	0.90	60.52
United Kingdom	5,407,428	128,960	2.40	192.95
Argentina	4,749,443	101,434	2.10	225.72
Colombia	4,621,260	115,831	2.50	230.1
Italy	4,284,332	127,864	3.00	212.06
Spain	4,100,222	81,096	2.00	172.26
Germany	3,751,253	91,369	2.40	109.91
Iran	3,501,079	86,966	2.50	104.89
Poland	2,881,355	75,212	2.60	198.08
Indonesia	2,832,755	72,489	2.60	26.79
Mexico	2,654,699	236,240	8.90	185.18
Ukraine	2,317,198	55,151	2.40	124.26
South Africa	2,283,880	66,676	2.90	113.86
Peru	2,092,125	195,047	9.30	599.95
Netherlands	1,816,689	18,060	1.00	104.2

*Source: WHO and JHU COVID-19 statistic. JUH, John Hopkins University*.

**Figure 2:**
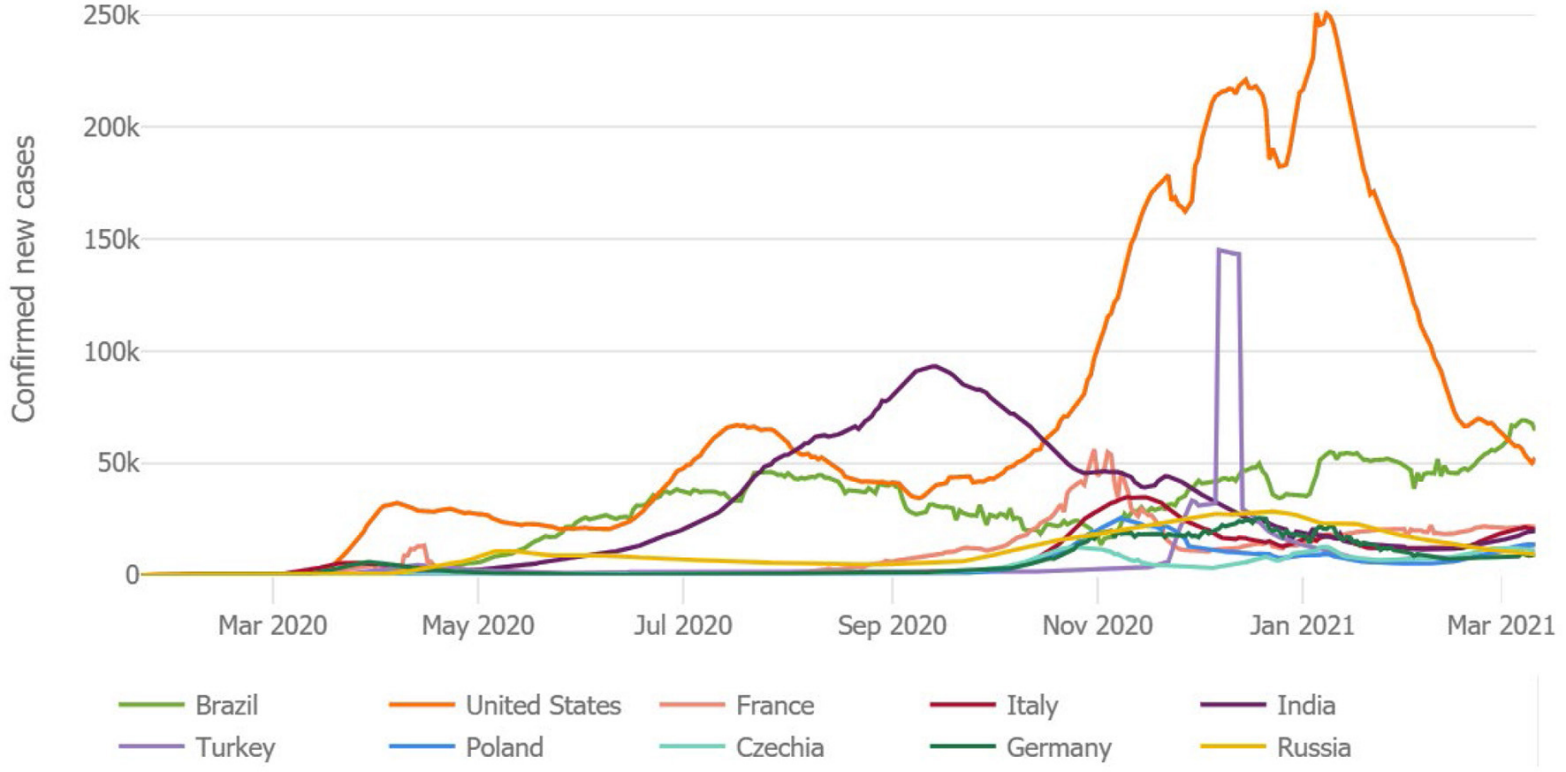
COVID-19: new confirmed positive cases (7-Day Moving Average) by March 12, 2021. Source: WHO and JHU COVID-19 information.

Figure 2 specifies the most affected countries worldwide. The US is still the most affected country with the most newly confirmed COVID-19 patients as of March 12, 2021. The second most affected nation is India, as indicated in Figure 2. The UK, Italy, Spain, France, and Brazil face a burden on health care systems. Many countries are incorporating preventive strategies to “flatten the COVID-19 cases curve.” Flattening the COVID-19 curve minimizes the quick spread of the disease and reduces new virus cases from 1 day to the next day. Reducing new COVID-19 cases helps prevent burdens on hospitals. When fewer COVID-19-positive cases emerge today than reported on the previous day, it shows that the country is successfully flattening the curve. A flattened curve shows a downward trend of recording new positive cases of the coronavirus. The 7-day moving average analysis visualizes recent COVID-19 cases, and this method reflects the rate of change. This study helps in offering valuable suggestions for the healthcare system to provide medical treatment to the patients, and it is helpful to lower the burden on healthcare systems around the world.

Table 2 indicates the COVID-19 verified positive cases in different countries around the globe. The WHO data reported that the USA remained the most affected country with 34,067,912 positive patients, 608,884 deaths, and 11,166,500 recovered patients. India was the second most affected country, with 10,746,183 confirmed patients, 154,274 total deaths, and 10,423,125 recoveries. Brazil reported 9,204,731 infected cases, 224,534 deaths, and 8,027,042 recoveries, as of February 1, 2021. There were 3,817,176 confirmed cases in the United Kingdom with 106,158 deaths, and France reported 3,197,114 patients with 76,057 deaths, Spain recorded 2,743,119 COVID-19-confirmed patients with 58,319 deaths. Italy declared 2,553,032 cases with 88,516 deaths, and the total number of recovered patients was 2,010,548 (see Table 3).

**Table 2:** The COVID-19 global cases in various affected countries as of July 18, 2021.

**Worldwide/country wise**	**Global cases**	**Global deaths**	**Global recoveries**
COVID-19 cases: February 1, 2021	102,964,429	2,227,900	57,049,238
COVID-19 cases: July 19, 2021	191,230,672	4,105,847	174,183,183
**Country-wise cases**	**Confirmed**	**Deaths**	**Case-fatality rate (%)**
United States	34,067,912	608,884	1.80
Brazil	19,342,448	541,266	2.80
India	31,106,065	413,609	1.30
Mexico	2,654,699	236,240	8.90
Peru	2,092,125	195,047	9.30
Russia	5,860,113	145,222	2.50
United Kingdom	5,407,428	128,960	2.40
Italy	4,284,332	127,864	3.00
Colombia	4,621,260	115,831	2.50
France	5,917,397	111,657	1.90
Argentina	4,749,443	101,434	2.10
Germany	3,751,253	91,369	2.40
Iran	3,501,079	86,966	2.50
Spain	4,100,222	81,096	2.00
Poland	2,881,355	75,212	2.60
Indonesia	2,832,755	72,489	2.60
South Africa	2,283,880	66,676	2.90
Ukraine	2,317,198	55,151	2.40
Turkey	5,522,039	50,488	0.90
Chile	1,598,481	34,403	2.20
Romania	1,081,588	34,252	3.20
Czechia	1,670,823	30,336	1.80
Hungary	808,725	30,015	3.70
Philippines	1,502,359	26,598	1.80
Pakistan	989,275	22,781	2.30

*Source: WHO and JHU CSSE COVID-19 Datasets*.

**Table 3:** COVID-19 cases and mortality of the most affected countries, as of July 18, 2021.

**Country**	**Confirmed**	**Deaths**	**Case-fatality**	**Deaths/100k**
	**cases**		**(%)**	**pop**.
Yemen	6,977	1,368	19.60	4.69
Peru	2,092,125	195,047	9.30	599.95
Mexico	2,654,699	236,240	8.90	185.18
Sudan	36,986	2,776	7.50	6.48
Syria	25,827	1,904	7.40	11.15
Egypt	283,636	16,439	5.80	16.38
Somalia	15,085	781	5.20	5.06
Taiwan	15,390	764	5.00	3.21
Bosnia and Herze^*^	205,285	9,666	4.70	292.82
China	104,257	4,848	4.70	0.35
Ecuador	475,215	21,933	4.60	126.24
Bulgaria	422,930	18,169	4.30	260.46
Afghanistan	137,853	5,983	4.30	15.73
Tanzania	509	21	4.10	0.04
Bolivia	461,714	17,443	3.80	151.51
Hungary	808,725	30,015	3.70	307.22
Comoros	4,009	147	3.70	17.28
Mali	14,509	530	3.70	2.7
North Macedonia	155,901	5,487	3.50	263.36
Chad	4,964	174	3.50	1.09
Niger	5,573	194	3.50	0.83
Eswatini	21,062	703	3.30	61.23
Antigua and Barbuda	1,268	42	3.30	43.25
Slovakia	392,034	12,524	3.20	229.63
Paraguay	444,427	14,230	3.20	202

*Source: WHO and JHU CSSE COVID-19 Datasets*;

* *Bosnia and Herzegovina*.

Table 3 shows the reported COVID-19 cases concerning case fatality rates in the leading and most affected countries globally. The WHO statistics showed that Yemen had the highest rate of case fatality, CFR = 19.60%, followed by Peru, CFR = 9.30% and Mexico = 8.90% as of July 19, 2021. Before this, the USA remained the most affected country. India declared the second highest positive cases of the coronavirus. Brazil reported 19,342,448 infected cases, 541,266 deaths, and a case fatality rate of 2.8%, as of July 18, 2021. There were 5,917,397 confirmed cases, over 111,657 deaths, and a case fatality rate of 1.9% in France. Russia acknowledged 5,860,113 established COVID-19 patients, 145,222 total deaths, and a case fatality rate of 2.5%. Turkey reported over 5,522,039 confirmed cases, a death toll of 50,488, and a case fatality rate of 0.90%. Similarly, the United Kingdom professed 5,407,428 total positive cases with 128,960 total deaths. Argentina reported a total of 4,749,443 cases of the coronavirus with a death toll of 101,434 and a case fatality ratio of 2.10%. Italy declared 4,284,332 cases with 127,864 deaths and a CFR of 3.00% as of July 18, 2021.

**Figure 3:**
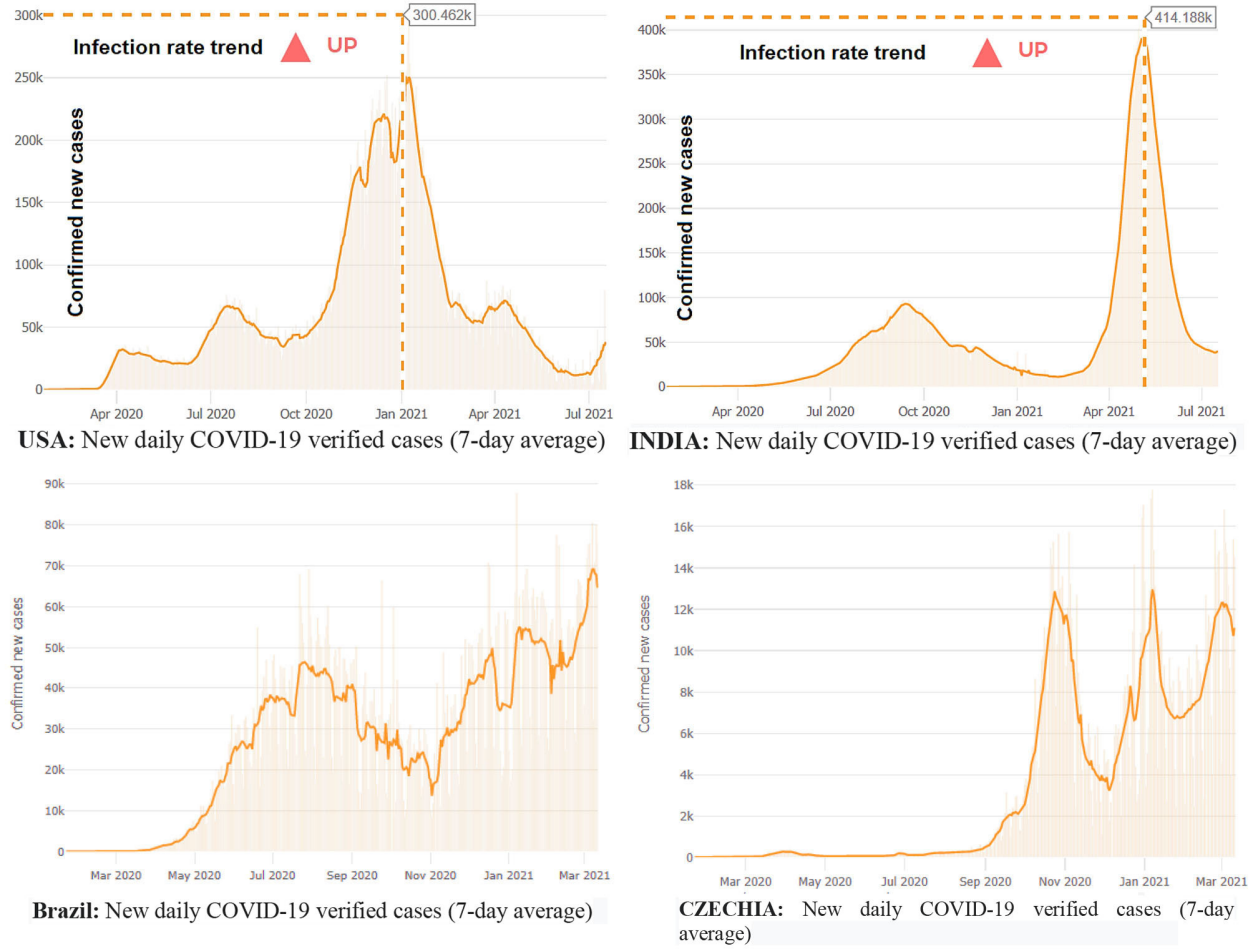
COVID-19 waves, positive cases in the USA, Brazil, India, and Czechia, July 18, 2021. Source: WHO and JHU CSSE data on COVID-19 disease.

The world is suffering from the damages of the new Delta variant, originating from B.1.617.2 lineage, a novel variant of SARS-CoV-2, which results in rapid transmission of the COVID-19 viral disease. Health experts first detected this new virus in India during the last quarter of 2020. Health experts investigated this new variant, WHO named it B.1.617, and then called it the Delta variant, on May 31, 2021. Public Health England (PHE) recorded a second attack ratio that remained 51–67% higher than the Alpha variant. The Delta variant is fatal and doubles the hospitalization cases of COVID-19 patients. The Delta variant has affected more than 80 countries as of July 12, 2021. The Delta variant massively struck India and brought a second wave at the beginning of February 2021. It resulted in the third wave in the United Kingdom (UK), Fiji, and South Africa. According to the WHO warning in July 2021, the Delta variant can bring new waves of COVID-19 in Africa, Asia, and Europe (67). The Center for Disease Control and Prevention (CDC) acknowledged Delta as a variant of international concern (68–71) (see Figure 3).

Virus transmission is still increasing in the US, Brazil, and India based on new tested and verified coronavirus infections cases on a 7-day average basis. The reported new cases of the viral disease have shown a rising trend in some countries, while some have declared a steep decline in new cases. For instance, the US has registered a decline in the number of new COVID-19 virus cases after January 2021. Likewise, Germany, France, and Czechia have reported an increasing trend of newly infected people, and Italy has announced an upsurge in new infections in February 2021. Many countries face new waves, such as first, second, third, and fourth waves, resulting from the ongoing pandemic COVID-19 worldwide. Europe has become the epicenter, and there were around 38 million cases of the virus as of March 2021. Brazil is also still very much part of the pandemic. Brazil confirmed the arrival of the disease on February 25, 2020. Since November 2020, Brazil has reported an increasing trend of new cases of COVID-19. By January 15, 2021, confirmed positive cases in Brazil exceeded 8,000,000, with a death toll exceeding 200,000. COVID-19 hit hardest in Brazil and made it the worst affected country in the world. Since then, Brazil has reported 11.4 million cases and more than 275,000 deaths. The USA reported the first confirmed case on January 22, 2020, and since then, the US has declared 29,347,338 cases, with a death toll of 532,590. There is a rising trend of the disease in India. The country has confirmed 11,333,728 COVID-19-positive patients and a death toll of 158,446 as of March 12, 2021. Yemen reported the highest case fatality (CFR = 25%) ratio, Mexico was the second highest (CFR = 9%), Syria reported a CFR of 6.7%, Sudan announced a CFR of 6.35), and Egypt reported a CFR of 5.9% as of March 12, 2021.

**Figure 4:**
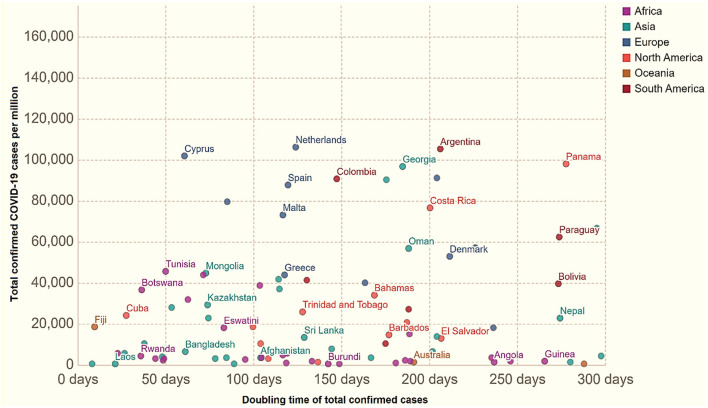
Total recorded COVID-19 cases/million vs. how quickly these figures double as of July 18, 2021.

The WHO and JHU datasets specified that Yemen had the highest CFR, 25%, in March 12, 2021. They reported 2,667 infected patients with 667 deaths, and a death rate/100K population of 2.34. Mexico remained the second highest with verified patients, and a CFR of 9%. Mexico reported 2,150,955 confirmed infected patients with a total death number of 193,152/100K population. Syria reported a CFR of 6.7%, and Sudan declared a 6.3% CFR. Table 4 displays the results for total cases per million vs. case doubling time. Figure 4 shows cases of COVID-19/million population vs. doubling the time of virus-infected patients in different regions across the world. Figure 4 stipulates the time of doubling patients from 0 to 50, 50 to 100, 100 to 150, 150 to 200, and 250 to 300 days in the countries and territories.

**Table 4:** COVID-19 global cases in the most affected countries, as of September 10, 2021.

**Country other**	**Total cases**	**New cases**	**Total deaths**	**New deaths**	**Total recovered**	**New recovered**	**Deaths/1M pop**	**Total tests**	**Population**
**World**	**222,783,507**	**539,809**	**4,599,186**	**8,604**	**199,279,326**	**591,567**	**590**	**-**	**-**
USA	41,239,828	115,776	669,483	919	31,544,460	83,240	2,009	597,328,264	333,304,877
India	33,095,450	38,130	441,443	368	32,256,552	39,090	316	533,189,348	1,396,087,038
Brazil	20,913,578	13,645	584,208	342	19,932,646	37,986	2,725	57,095,219	214,351,247
UK	7,055,262	37,148	133,483	209	5,667,508	30,695	1,954	277,951,515	68,309,023
Russia	7,047,880	17,425	188,785	795	6,302,250	17,243	1,293	181,500,000	146,008,739
France	6,854,028	14,534	115,159	152	6,418,376	28,303	1,760	129,298,105	65,444,916
Turkey	6,542,654	23,638	58,651	274	5,994,394	34,402	687	78,518,387	85,414,656
Argentina	5,211,801	4,106	112,851	178	4,909,453	3,610	2,470	22,648,977	45,687,431
Iran	5,184,124	27,138	111,892	635	4,422,740	28,657	1,312	29,227,907	85,266,654
Colombia	4,921,410	1,637	125,378	47	4,756,976	2,321	2,433	24,493,353	51,525,677
Spain	4,892,640	5,246	85,066	138	4,503,204	21,561	1,819	60,618,810	46,776,274
Italy	4,579,500	4,718	129,638	71	4,316,077	6,877	2,148	85,973,261	60,356,270
Indonesia	4,140,634	7,201	137,156	683	3,864,848	14,159	495	33,641,037	276,951,672
Germany	4,029,849	9,276	92,949	47	3,783,800	8,300	1,105	70,379,237	84,101,825
Mexico	3,433,511	5,127	263,470	330	2,782,729	13,783	2,018	9,971,374	130,538,296

*Source: WHO and JHU CSSE COVID-19 Data. Yellow highlight shows new cases, red highlight new deaths, and green highlight specifies new recovered cases in selected countries*.

**Figure 5:**
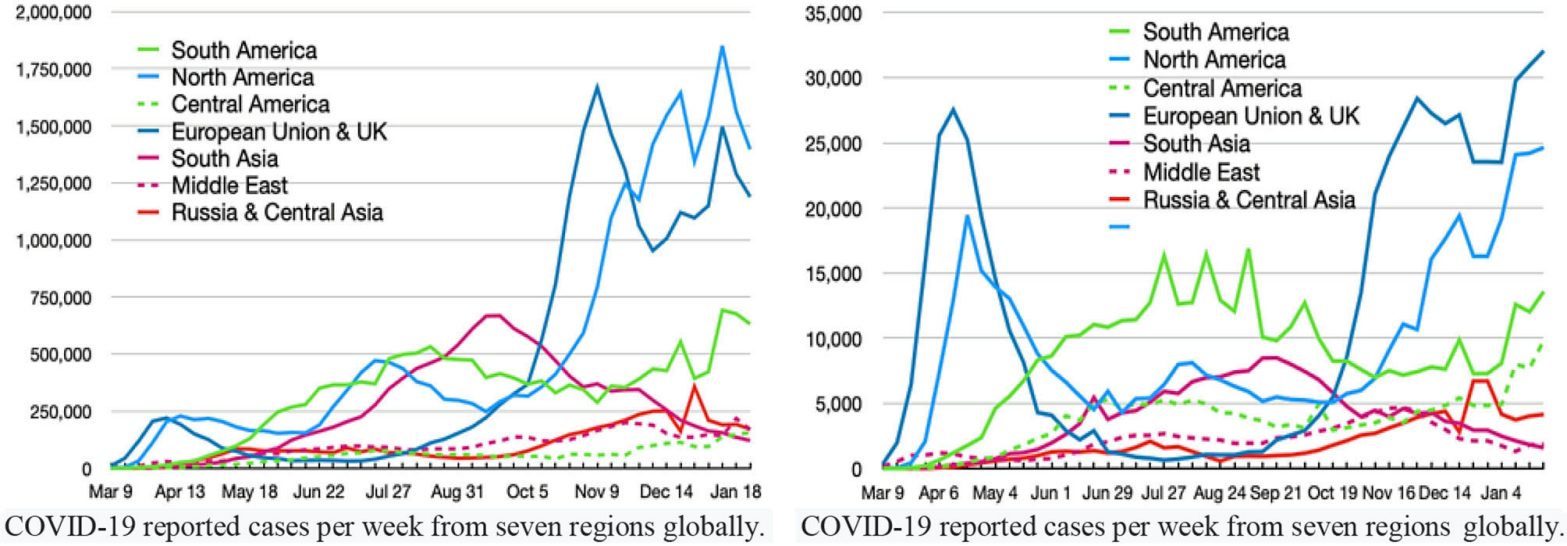
COVID-19 identified cases vs. deaths due to coronavirus in the top seven regions, globally. Source: JHU and WHO dashboard.

Figure 5 reports on COVID-19 identified cases vs. deaths due to the coronavirus in the top seven regions, as of January 23, 2020, worldwide. The arrival of the pandemic has also immensely affected Pakistan's economy (72, 73). The pandemic influenced all the key factors and damaged economic activities. As of September 9, 2021, there were 1,194,198 verified patients, 26,497 total deaths (2.2%), and 1,076,112 recoveries in Pakistan. The pandemic posed enormous economic, health, social and political, and environmental issues in society. The pandemic's second, third, and fourth waves in Pakistan were potent and fatal, resulting in thousands of deaths. Pakistan reported 920,066 verified COVID-19 patients, a death toll of 22,811 (2.3%), and the recovery rate remained 92.8% as of July 19, 2021. As of March 12, 2021, China had a 4.8% case fatality rate, followed by Australia: 3.1%, the United Kingdom: 2.6%, Africa: 2.6%, Europe: 2.4%, the whole world: 2.2%, Asia: 1.7%, and the United States of America: 1.7%.

Facts stipulate that the European Union and the United Kingdom reported 27,385,890 total infected cases, 53,137 cases per million, with a death toll of 680,930, and deaths per million of 1,321. North America declared 29,880,184 total cases, 81,018 cases per million, 542,230 deaths, and 1,470 deaths per million. Similarly, South America recorded 12,809,782 COVID-19 cases, 43,396 cases per million, 482,707 deaths, and 1,123 deaths per million. South Asia reported 12,809,782 total positive cases, 6,900 cases per million, 186,009 deaths, and 100 deaths per million. Central America recorded 3,115,633 infected cases, 17,341 cases per million, a death toll of 212,838, and 1,185 deaths per million. The Middle East declared 5,712,789 total cases, 21,889 cases per million, a total death toll of 105,482, and 404 fatalities per million.

Table 4 shows the number of global infected patients, total deaths, total recoveries, and new cases and deaths as of September 10, 2021. It shows a detailed analysis of the COVID-19 global spread, infections, recoveries, deaths, and critical patients.

**Figure 6:**
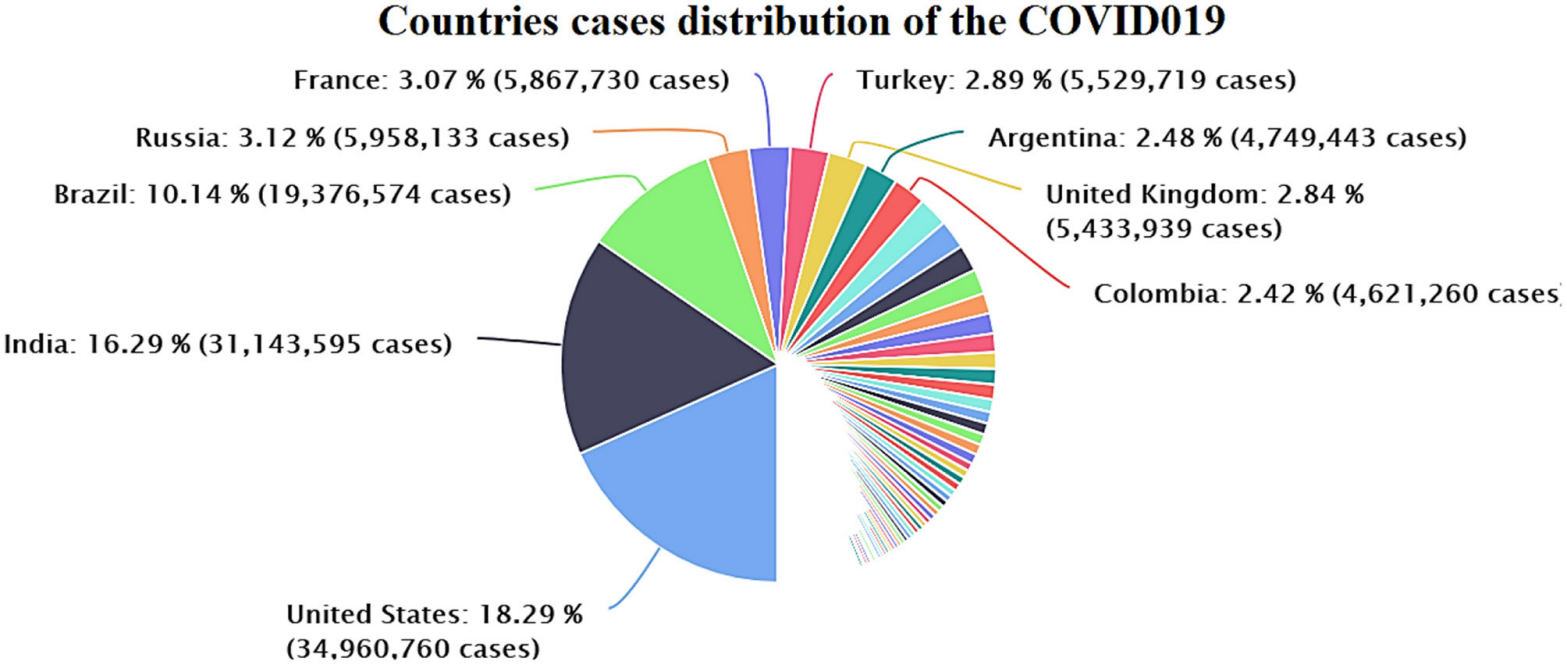
Distribution of the cases in various countries worldwide (July 18, 2021). Source: Data provided by World Health Organization related to the coronavirus (COVID-19).

Table 5 presents the global scenario of COVID-19 transmission and infected patients, total cases, total deaths, total recoveries, and new cases and deaths worldwide. It shows a detailed analysis of the COVID-19 global spread, infections, recoveries, deaths, and critical patients in the world as of July 19, 2021. The pandemic has posed global, regional, and domestic-level health challenges worldwide (5, 74–79). Figure 6 shows the most affected countries that reported the highest number of verified and tested patients of COVID-19. See Table 4 and Figure 6 on the global health crisis and number of infected people worldwide.

**Table 5:** Worldwide COVID-19 cases and the top 30 most affected countries, as of July 19, 2021.

**Worldwide countries**	**Total cases**	**New cases**	**Total deaths**	**New deaths**	**Total recovered**	**Active cases**	**Serious, critical**	**Total cases/1M pop**	**Deaths/1M pop**	**Total tests**	**Population of country**
**Worldwide cases**	**192,209,053**	**491,794**	**4,124,198**	**8,010**	**174,910,919**	**13,173,936**	**81,774**	**24,659**	**529.1**	**N/A**	**7,880,702,437**
USA	35,077,689	41,098	625,244	220	29,434,213	5,018,232	6,283	105,326	1,877	518,717,489	333,040,210
India	31,215,142	42,123	418,511	489	30,383,001	413,630	8,944	22,389	300	447,341,133	1,394,235,023
Brazil	19,419,741	27,896	544,302	1,425	18,124,621	750,818	8,318	90,685	2,542	54,786,381	214,145,148
Russia	6,006,536	23,770	149,922	784	5,382,213	474,401	2,300	41,141	1,027	159,900,000	146,000,220
France	5,890,062	18,181	111,525	33	*5,663,809*	114,728	876	90,027	1,705	99,504,244	65,425,241
Turkey	5,546,166	8,780	50,650	46	5,395,300	100,216	543	65,027	594	65,300,191	85,290,746
UK	5,519,602	46,558	128,823	96	4,411,839	978,940	611	80,861	1,887	234,640,904	68,260,406
Argentina	4,784,219	15,077	102,381	426	4,420,995	260,843	4,643	104,846	2,244	18,420,915	45,630,830
Colombia	4,668,750	12,829	117,131	378	4,422,866	128,753	8,155	90,741	2,277	21,744,142	51,451,630
Italy	4,293,083	3,558	127,884	10	4,115,889	49,310	165	71,115	2,118	75,100,357	60,368,368
Spain	4,189,136	27,286	81,148	29	3,676,323	431,665	1,116	89,562	1,735	55,184,196	46,773,809
Germany	3,754,828	1,626	91,938	30	3,641,000	21,890	361	44,666	1,094	65,845,568	84,065,322
Iran	3,576,148	27,444	87,624	250	3,168,834	319,690	4,422	42,013	1,029	24,892,912	85,119,790
Indonesia	2,950,058	38,325	76,200	1,280	2,323,666	550,192	13,550	10,667	276	23,719,489	276,556,785
Poland	2,881,594	104	75,219	4	2,653,034	153,341	60	76,227	1,990	18,138,390	37,803,040
Mexico	2,664,444	5,307	236,469	138	2,095,953	332,022	4,798	20,440	1,814	7,960,939	130,353,354
South Africa	2,311,232	8,928	67,676	596	2,085,119	158,437	546	38,461	1,126	14,310,166	60,092,502
Netherlands	1,814,143	6,699	17,783	5	*1,661,205*	135,155	95	105,629	1,035	15,472,423	17,174,700
Philippines	1,517,903	4,516	26,844	58	1,444,253	46,806	2,031	13,661	242	16,042,300	111,109,628
Iraq	1,510,517	8,922	17,951	59	1,372,158	120,408	720	36,693	436	12,587,768	41,166,304
Canada	1,424,220	342	26,508	4	1,393,117	4,595	264	37,392	696	37,947,701	38,089,322
Belgium	1,107,208	1,330	25,213	2	1,051,372	30,623	88	95,100	2,166	16,330,887	11,642,587
Romania	1,081,773	95	34,258	3	1,046,881	634	36	56,629	1,793	10,257,994	19,102,669
Pakistan	993,872	2,145	22,848	37	921,095	49,929	2,697	4,410	101	15,484,282	225,380,778
Malaysia	939,899	12,366	7,241	93	798,955	133,703	924	28,655	221	16,622,925	32,800,653

*Source: WHO and JHU CSSE COVID-19 Data. Yellow highlight shows new cases and red highlight new deaths*.

Figure 6 demonstrates the confirmed infected patients of COVID-19 in different countries worldwide. The WHO statistics reported the confirmed total cases and death toll, cases/million, number of deaths/million, total population by different regions, absolute facts and figures, and ratio per million inhabitants. US patients make up 18.29% (34,960,760) of the global infected cases (191,324,829). India reports 16.29%, a total of 31,143,595 as of July 19, 2021. There were over 223.475 million COVID-19 cases, 4.611 million deaths, and more than 200.0111 million recovered patients globally, as of September 9, 2021.

## Social Media Use and Health Behavior

The coronavirus has affected all spheres of society, leading to reshaping human attitudes toward life (5, 78–81). The coronavirus outbreak has influenced human behaviors, leading to people relying on updates through social media platforms and other reliable sources. Social media tools help provide the latest news and health-related updates amid the COVID-19 crisis (82). The different waves of the current pandemic have created a massive burden and caused health risks for human beings, leading to an increase in the usage of social media applications as people seek the latest news about preventive measures against the COVID-19 disease. People look for precise and correct information related to their health, which has led to more use of platforms of media technologies to stay connected with family members, peers, and friends (83–86). People seek accurate information related to viral disease, and social media use has helped people find public communication to follow preventive measures (87). The coronavirus crisis has dramatically influenced human preventive behaviors and life patterns to safeguard themselves against the COVID-19 virus globally (88). The global COVID-19 health crisis has promoted health behaviors, and people have adopted preventive measures such as social distancing, avoiding social gatherings, washing hands, and using face masks (89, 90). Humans health behavior theories help describe the health behaviors change model (91). The HBM (health behavior model) helps understand the supportive interventions and strategies for promoting healthy behaviors. For instance, adherence and following SOPs and medical treatment to combat this fatal viral disease (92). Thus, the health behavior measuring model provides valuable information. It interprets that humans are involved in health-related behaviors when they perceive vulnerability to a fatal and contagious viral illness that can lead to dangerous health-related adverse results. This type of preventive health-related measure help people to combat viral infectious disease, and it helps to overcome the barriers (93). The health crisis of COVID-19 has influenced individuals' emotions and their life patterns' quality (94). Health experts need managerial skills to handle health emergencies in a crisis (95).

This present paper primarily focuses on examining the crucial effects of social media and web-based technological applications in the crisis of the current pandemic. The study investigates how social media can help in the COVID-19 crisis in providing accurate information related to this viral disease to allow people to remain safe by following preventive measures. Social media technology is vital for responding to and adopting practical actions to manage COVID-19 damages and health crises. The public receives updated, reliable, and credible health-related material by using social media tools. Accordingly, social media has greatly helped all spheres of life in society get back to the next normal. Business activities have become possible with work from home policies. Universities and educational institutions have adopted online learning methods in response to restrictions on face-to-face learning imposed by the governments to minimize the damage of the COVID-19 outbreak (96). The online and virtual learning methods helped reduce student and teacher stress and offered them relief in the stressful situation of the COVID-19 crisis. This study examines how social media technological applications have helped people respond to the crisis environment and minimize the damage of COVID-19 that has resulted in mental health issues worldwide. Social media applications can improve social interaction and harness contacts and social support (97). The pandemic has changed socialization patterns, and people use online tools and social media for communication with friends and relatives via video calls, telephone, and other social media apps. Interventions help combat the transmission of COVID-19, and social media technology tools are effective in changing perception and behavior amid the COVID-19 health crisis (28).

## Environmental Impacts

According to expert estimations, shutdowns of transportation and economic activities in this pandemic will lower global emissions for the first time since the world financial crisis of 2008 (84, 98–101). The pandemic has affected demand for renewable energy consumption, trade on environmental quality, and human capital (102–104). The pandemic has affected global and regional progress and disrupted economic activities on micro and macro levels (105–108). Carbon Brief analysis specifies that the emergence of the ongoing COVID-19 outbreak might decrease carbon dioxide emissions by up to 1,600 million tons during the year 2020 and onwards. This decrease in emission equals 5.50% of 2019's total emissions worldwide. It equals removing 3.47 billion passengers from the highways and roads for 1 year, according to the Greenhouse Gas Equivalence Calculator (GGEC) measurement, which is assessed by the Environmental Protection Agency (EPA). The decline in air pollution has resulted in substantial health benefits; however, it has not alleviated the catastrophic consequences of the COVID-19 pandemic. It has resulted in over 191.085 million confirmed COVID-19 patients. As of July 18, there were over 191,084,631 verified coronavirus patients, more than 4,103,306 deaths and 174,041,354 recovered patients worldwide (109).

## Crisis Management and Healthcare Systems

Besides, the emergence of this transmittable disease speedily instigated significant disruption to societies, healthcare systems, and global economies. The COVID-19 outbreak posed global challenges and problems of economic crisis management, which are still unfolding. This article explores the effects and strategic retorts on the protective actions for combating the viral disease COVID-19 and its adverse consequences on social, environmental, economic, and health factors. The study primarily focused on challenges and crises on the economy, business activity, healthcare burden, and government support for society. This research suggests that intervention strategies control the rapid spread of COVID-19 through a hands-on crisis management system, and the healthcare system will quickly resume normalcy. Through government support, the global economy will revive through scientific contributions and collaborations, including social science and the business industry (110). Health crisis management is a challenging and complex subject. Global economic crises necessitate institutional, organizational, and individual responses at large-scale with an effective direction by linking various approaches based on interdisciplinary and multidisciplinary tactics to handle this vital issue. Each crisis indicates a positive aspect inheritance to perceive and respond to a challenge or a crisis. Resilience helps deal with a challenge or a crisis effectively. Economic crisis management in a challenging situation provides opportunities to capitalize on new openings and economic horizons. Organizations, business managers, and financial experts need adequate and innovative organizational skills, innovative business plans, and entrepreneurship.

## NPIs (Non-Pharmaceutical Interventions) in the Absence of COVID-19 Vaccines

Non-pharmaceutical interventions are the methods to combat the ongoing COVID-19 pandemic's adverse impact, apart from taking medicines, or getting vaccinated, which communities and ordinary people can take as preventive measures to help minimize the rapid spread of the infectious disease. The past studies on the Spanish influenza 1918 (H1N1) pandemic showed that close settings, army barracks, college and university dormitories, early patients' identification, and isolation of the infected patients did not stop the infection transmission (18). However, it was helpful to decrease rapid spread, especially with restrictions on social gatherings and travel bans to and from the adjacent communities (111). Health experts take NPIs as the mitigation strategies of communities to slow and reduce the pandemic's quick transmission. When a novel virus rapidly spread in communities and infects people, causing contagious diseases worldwide, it becomes a pandemic flu as per the declaration of the WHO. The ongoing virus is a novel disease, and human populations have little or no strong immunity against the pandemic. It has massively infected global communities.

This new virus, COVID-19, quickly spreads among people and infects people who make close contact with infected patients. In this panic situation, the WHO and health professionals have declared NPIs among the best possible preventive strategies for controlling the quick transmission of the pandemic when effective medications and vaccines are not yet available to general populations at large scale. The literature documented that NPIs are significantly practical and helpful to reduce the quick transmission of the deadliest diseases. Implementing the social distancing strategy is incredibly effective and beneficial, more so than other NPIs, to contain the rapid spread of the coronavirus. Two or more synchronous NPIs are more productive and useful than a single NPI strategy. The literature indicated that NPIs help contains the rapid spread of the COVID-19 transmission significantly. Based on the above debate, social distancing and implementing two or more NPI strategies can significantly contain the quick spread among people. These strategies should be the priority in the ongoing panic situation of COVID-19.

This study has proposed two viable, practical rudimentary strategies: (1) a mitigation strategy which principally focuses on preventive measures through social distancing and lockdown to control and minimize COVID-19's rapid transmission in human society. However, it is not considered helpful in preventing the spread of the epidemic. This strategy is useful in sustaining or reducing the peak burdens on healthcare systems. (2) The suppression model intended to reverse the epidemic's development by lowering infections from higher or lower ratios or permanently maintaining the suppression policy. Each approach has the challenge to gain positive results. This article explains that the best possible and useful mitigation strategy is combining quarantine for suspected patients and maintaining social distancing/separation of aged people and individuals facing higher risks of COVID-19. It helps reduce the peak burdens on healthcare systems by two-thirds (67%), and it decreases the rate of deaths (50%) by half (112). On the contrary, the mitigation policy can lower the pressure on the healthcare systems to facilitate the patients. It helps minimize the rate of casualties, as the mitigation strategy reduces the probability of rapid spread of the disease. Many countries have opted for these strategies to control the rapid spread of contagious diseases, and they have achieved their goals by minimizing infections in the general population (113).

In the current global situation, suppression recommends social distancing to minimize the spread of the COVID-19 disease. Isolation for suspected individuals and household quarantining is helpful for the relatives of the alleged cases of COVID-19. It helps to prioritize health needs among people (114–118). There could be a recommendation for the closure of universities, colleges, and schools worldwide. However, such a restricted and upset environment might create deleterious impacts on individuals' mental health because of social distance and the absence of face-to-face interactions (119–123). This article describes the implications of resilience, economic crisis management, global health, and economic challenges in combatting the adverse effects of the pandemic. This study focuses on global challenges with a case study of Pakistan. This study aims to discuss the negative impacts of the ongoing COVID-19 outbreak on global economies, business industries, and how governments support societies and business sectors to minimize the disruptions through implications for global economic and healthcare provision disruptions. This study suggests that intervention strategies control the rapid spread of COVID-19 with hands-on crisis management measures, and the healthcare system will resume normal conditions quickly. Global economies will revitalize scientific contributions and collaborations, including social science and business industries, through government support.

## Conclusion

The world has encountered the massive shock of the ongoing waves of the pandemic, and it still continues to affect people worldwide. Experience documented in the 1918 Spanish influenza (H1N1) pandemic showed that timely patient identification and isolation, including restrictions on social ties and travel bans from adjacent communities, did not stop disease transmission but helped decrease the virus spread. Lessons from past pandemics have showed us that public health interventions such implementing closure of educational institutes (schools, colleges, and universities), swimming pools, restaurants, and other public places, canceling sports events, and disinfecting buses, taxis, and public areas are very beneficial. The majority of the individuals and communities strictly followed preventive measures, used masks in public places, and washed hands frequently. A high percentage of people avoided unnecessary social gatherings and mixing among people. Social distancing dramatically decreased virus transmission among communities.

The World Health Organization suggested preventive measures be taken in conjunction with this global health emergency to combat COVID-19. The general relaxation of social distancing and lockdown policies has led to a higher number of recorded incidents, infections, and more deaths worldwide. Remarkably, the second wave has been more potent and lethal in Europe, and the USA and governments are under pressure to provide health care facilities to patients. This pandemic has already posed significant impacts on societies, individuals, healthcare systems, and human well-being. Since the advent of the epidemic, a wide range of challenges and economic crisis management issues have developed for everybody worldwide. Thus, the coronavirus crisis will have massive adverse effects that are not yet visible in the long run. The practical solutions, knowledge, and various studies will provide insights to limit and counter the adverse consequences of COVID-19. However, each crisis offers some aspects to capitalize on learning and seeking knowledge based on positive and negative experiences.

This study explains that non-pharmaceutical interventions are useful in responding to the COVID-19 challenges and economic crisis to manage the spillover impacts on global mental health disasters and financial emergencies. The various combinations of NPIs help to drastically decrease the virus attacks and quick transmission of the contagious disease among healthy communities worldwide. The suppression model introduced in China has been helpful to minimize the rapid spread of COVID-19, as the Government of China strictly implemented restrictions on social gatherings. Pakistan applied a mixed method of NPIs and gained very productive results. Initially, Pakistan implemented both lockdown and mitigation strategies and later implemented a smart lockdown, which identified highly infected communities and areas. The WHO appreciated the smart lockdown strategy, as it helped to resume life to standard settings gradually. In the absence of effective vaccines, NPIs help combat the COVID-19 health crisis worldwide.

## Author Contributions

JA and RM contributed to study design. YZ, AD, MEB, MAS, and JA had edited the original manuscript before submission and editing and approving the final edited manuscript. JA contributed to idea conceptualization, project administration, supervision, contributed to writing the study abstract, discussion, implications, and review. RM contributed to methodology. YZ, AD, MEB, and MAS contributed to study cost, literature, checked writing, substantially revised, and critically reviewed the article. AD critically reviewed the manuscript and contributed to APC. All authors did significant changes introduced at the proofing stage, contributed to the paper, and approved the submitted version for publication.

## Funding

The publication of this study was partially funded by Politehnica University of Timisoara (UPT), Romania. This work was funded by the Deanship of Scientific Research (DSR), King Abdulaziz University Jeddah under Grant (No. DF-689-120-1441). The authors acknowledged the Deanship of Scientific Research and the Politehnica University of Timisoara with thanks for their technical and financial support.

## Conflict of Interest

The authors declare that the research was conducted in the absence of any commercial or financial relationships that could be construed as a potential conflict of interest. The reviewer MB declared a shared affiliation, with no collaboration, with the author JA at the time of the review. The reviewer SB declared a shared affiliation, with no collaboration, with the author RM at the time of the review.

## Publisher's Note

All claims expressed in this article are solely those of the authors and do not necessarily represent those of their affiliated organizations, or those of the publisher, the editors and the reviewers. Any product that may be evaluated in this article, or claim that may be made by its manufacturer, is not guaranteed or endorsed by the publisher.

## References

[1] Tosato M Carfi A Martis I Pais C Ciciarello F Rota E. Prevalence and predictors of persistence of COVID-19 symptoms in older adults: a single-center study. J Am Med Dir Assoc. (2021) 22:1840–4. 10.1016/j.jamda.2021.07.003 34352201 PMC8286874

[2] Demichev V Tober-Lau P Lemke O Nazarenko T Thibeault C Whitwell H. A time-resolved proteomic and prognostic map of COVID-19. Cell Syst. (2021) 12:780–94. 10.1016/j.cels.2021.05.005 34139154 PMC8201874

[3] Andraus M Thorpe J Tai XY Ashby S Hallab A Ding D. Impact of the COVID-19 pandemic on people with epilepsy: findings from the Brazilian arm of the COV-E study. Epilepsy Behav. (2021) 123:108261. 10.1016/j.yebeh.2021.108261 34481281 PMC8457887

[4] Grant MC Geoghegan L Arbyn M Mohammed Z McGuinness L Clarke EL. The prevalence of symptoms in 24,410 adults infected by the novel coronavirus (SARS-CoV-2; COVID-19): a systematic review and meta-analysis of 148 studies from 9 countries. PLoS ONE. (2020) 15:e0234765. 10.1371/journal.pone.0234765 32574165 PMC7310678

[5] Wang C Wang D Abbas J Duan K Mubeen R. Global financial crisis, smart lockdown strategies, and the COVID-19 spillover impacts: a global perspective implications from Southeast Asia. Front Psychiatry. (2021) 12:643783. 10.3389/fpsyt.2021.643783 34539457 PMC8446391

[6] Ainsworth M Andersson M Auckland K Baillie JK Barnes E Beer S. Performance characteristics of five immunoassays for SARS-CoV-2: a head-to-head benchmark comparison. Lancet Infect Dis. (2020) 20:1390–400. 10.1016/S1473-3099(20)30634-4 32979318 PMC7511171

[7] Santander-Gordon D Iturralde GA Freire-Paspuel B Zambrano-Mila MS Morales-Jadan D Vallejo-Janeta PA. Crucial contribution of the universities to SARS-CoV-2 surveillance in Ecuador: lessons for developing countries. One Health. (2021) 13:100267. 10.1016/j.onehlt.2021.100267 34056057 PMC8146272

[8] Liu Z Skowron K Grudlewska-Buda K Wiktorczyk-Kapischke N. The existence, spread, and strategies for environmental monitoring and control of SARS-CoV-2 in environmental media. Sci Tot Environ. (2021) 795:148949. 10.1016/j.scitotenv.2021.148949 34252782 PMC8262394

[9] Gorbalenya AE Baker SC Baric RS de Groot RJ Drosten C Gulyaeva AA. The species Severe acute respiratory syndrome- related coronavirus: classifying 2019-nCoV and naming it SARS-CoV-2. Nat Microbiol. (2020) 5:536–44. 10.1038/s41564-020-0695-z 32123347 PMC7095448

[10] Gan ES Syenina A Linster M Ng B Zhang SL Watanabe S. A mouse model of lethal respiratory dysfunction for SARS-CoV-2 infection. Antiviral Res. (2021) 193:105138. 10.1016/j.antiviral.2021.105138 34246735 PMC8264561

[11] Wang L Didelot X Bi Y Gao GF. Assessing the extent of community spread caused by mink-derived SARS-CoV-2 variants. Innovation. (2021) 2:100128. 10.1016/j.xinn.2021.100128 34124706 PMC8182980

[12] Kanbur N Barral R Efevbera Y Kelley MA Svetaz MV Miller E. Call to action against femicide: illuminating a shadow pandemic as a global public health emergency. J Adolesc Health. (2021) 68:443–6. 10.1016/j.jadohealth.2020.11.022 33431250

[13] Li C Sotomayor-Castillo C Nahidi S Kuznetsov S Considine J Curtis K. Emergency clinicians' knowledge, preparedness and experiences of managing COVID-19 during the 2020 global pandemic in Australian healthcare settings. Australas Emerg Care. (2021) 24: 186–196. 10.1016/j.auec.2021.03.008 34120888 PMC7998048

[14] Akintunde TY Musa TH Musa HH Musa IH Chen S Ibrahim E. Bibliometric analysis of global scientific literature on effects of COVID-19 pandemic on mental health. Asian J Psychiatr. (2021) 63:102753. 10.1016/j.ajp.2021.102753 34280888 PMC9760346

[15] Ji Y Shao J Tao B Song H Li Z Wang J. Are we ready to deal with a global COVID-19 pandemic? Rethinking countries' capacity based on the Global Health Security Index. Int J Infect Dis. (2021) 106:289–94. 10.1016/j.ijid.2021.03.089 33823282 PMC8019240

[16] Van Hout MC Wells JSG. The right to health, public health, and COVID-19: a discourse on the importance of the enforcement of humanitarian and human rights law in conflict settings for the future management of zoonotic pandemic diseases. Public Health. (2021) 192:3–7. 10.1016/j.puhe.2021.01.001 33601306 PMC7834498

[17] van Schalkwyk MC Maani N McKee M. Public health emergency or opportunity to profit? The two faces of the COVID-19 pandemic. Lancet Diabetes Endocrinol. (2021) 9:61–3. 10.1016/S2213-8587(21)00001-2 33417836 PMC9765138

[18] Lau K Dorigatti I Miraldo M Hauck K. SARIMA-modelled greater severity and mortality during the 2010/11 post-pandemic influenza season compared to the (2009). H1N1 pandemic in English hospitals. Int J Infect Dis. (2021) 105:161–71. 10.1016/j.ijid.2021.01.070 33548552

[19] Spreeuwenberg P Kroneman M Paget J. Reassessing the Global Mortality Burden of the 1918. Influenza pandemic. Am J Epidemiol. (2018) 187:2561–7. 10.1093/aje/kwy191 30202996 PMC7314216

[20] Thorlund K Dron L Park J Hsu G Forrest JI Mills EJ. A real-time dashboard of clinical trials for COVID-19. Lancet Digit Health. (2020) 2:e286–7. 10.1016/S2589-7500(20)30086-8 32363333 PMC7195288

[21] Dong E Du H Gardner L. An interactive web-based dashboard to track COVID-19 in real- time. Lancet Infect Dis. (2020) 20:533–4. 10.1016/S1473-3099(20)30120-1 32087114 PMC7159018

[22] Sanchez Turet M Gonzalez Sastre F Sabater Tobella J. [Effects of early malnutrition on the maturity of the nervous system. IV. Changes in the morphological and neurological development]. Arch Neurobiol. (1978) 41:177–88. 100076

[23] Su Z McDonnell D Cheshmehzangi A Abbas J Li X Cai Y. The promise and perils of Unit 731 data to advance COVID-19 research. BMJ Global Health. (2021) 6:e004772. 10.1136/bmjgh-2020-004772 34016575 PMC8141376

[24] NeJhaddadgar N Ziapour A Zakkipour G Abbas J Abolfathi M Shabani M. Effectiveness of telephone-based screening and triage during COVID-19 outbreak in the promoted primary healthcare system: a case study in Ardabil province, Iran. Z Gesundh Wiss. (2020) 29:1–6. 10.1007/s10389-020-01407-8 33224715 PMC7665795

[25] Lebni JY Toghroli R Abbas J Kianipour N NeJhaddadgar N Salahshoor MR. Nurses' work-related quality of life and its influencing demographic factors at a public hospital in Western Iran: a cross-sectional study. Int Q Community Health Educ. (2021) 42:37–45. 10.1177/0272684X20972838 33201756

[26] Selvey LA Antao C Hall R. Entry screening for infectious diseases in humans. Emerg Infect Dis. (2015) 21:197–201. 10.3201/eid2102.131610 25625224 PMC4313627

[27] Lee A. Wuhan novel coronavirus (COVID-19): why global control is challenging? Public Health. (2020) 179:A1–2. 10.1016/j.puhe.2020.02.001 32111295 PMC7130979

[28] Thornicroft G Mehta N Clement S Evans-Lacko S Doherty M Rose D. Evidence for effective interventions to reduce mental-health-related stigma and discrimination. Lancet. (2016) 387:1123–32. 10.1016/S0140-6736(15)00298-6 26410341

[29] Bénézit F Le Turnier P Declerck C Paillé C Revest M Dubée V. Utility of hyposmia and hypogeusia for the diagnosis of COVID-19. Lancet Infect Dis. (2020) 20:1014–5. 10.1016/S1473-3099(20)30297-8 32304632 PMC7159866

[30] Khosravani V Aardema F Samimi Ardestani SM Sharifi Bastan F. The impact of the coronavirus pandemic on specific symptom dimensions and severity in OCD: a comparison before and during COVID-19 in the context of stress responses. J Obsessive Compuls Relat Disord. (2021) 29:100626. 10.1016/j.jocrd.2021.100626 33520614 PMC7834974

[31] Nogrady B. What does the data say about asymptomatic COVID infections. Nature. (2020) 587:534–5. 10.1038/d41586-020-03141-3 33214725

[32] Gao Z Xu Y Sun C Wang X Guo Y Qiu S. A systematic review of asymptomatic infections with COVID-19. J Microbiol Immunol Infect. (2020) 54:12–6. 10.1016/j.jmii.2020.05.001 32425996 PMC7227597

[33] Furukawa NW Brooks JT Sobel J. Evidence supporting transmission of severe acute respiratory syndrome coronavirus 2 while presymptomatic or asymptomatic. Emerg Infect Dis. (2020) 26:e201595. 10.3201/eid2607.201595 32364890 PMC7323549

[34] Gandhi RT Lynch JB Del Rio C. Mild or moderate covid-19. N Engl J Med. (2020) 383:1757–66. 10.1056/NEJMcp2009249 32329974

[35] Wiersinga WJ Rhodes A Cheng AC Peacock SJ Prescott HC. Pathophysiology, transmission, diagnosis, and treatment of coronavirus disease 2019. (COVID-19): a review. JAMA. (2020 324:782–93. 10.1001/jama.2020.12839 32648899

[36] Zhang Y Lange KW. Coronavirus disease (2019). (COVID-19) and global mental health. Global Health J. (2021) 5:31–6. 10.1016/j.glohj.2021.02.004 33614179 PMC7881705

[37] Hamer M Kivimaki M Gale CR Batty GD. Lifestyle risk factors, inflammatory mechanisms, and COVID-19 hospitalization: a community-based cohort study of 387,109 adults in the UK. Brain Behav Immun. (2020) 87:184–7. 10.1016/j.bbi.2020.05.059 32454138 PMC7245300

[38] Maqsood A Abbas J Rehman G Mubeen R. The paradigm shift for educational system continuance in the advent of COVID-19 pandemic: mental health challenges and reflections. Curr Res Behav Sci. (2021) 2:100011. 10.1016/j.crbeha.2020.100011 PMC7832654 38620741

[39] John A Ali K Marsh H Reddy PH. Can a healthy lifestyle reduce the disease progression of Alzheimer's during a global pandemic of COVID-19? Ageing Res Rev. (2021) 70:101406. 10.1016/j.arr.2021.101406 34242809 PMC8259043

[40] Chik AHS Glier MB Servos M Mangat CS Pang X-L Qiu Y. Comparison of approaches to quantify SARS-CoV- 2 in wastewater using RT-qPCR: results and implications from a collaborative inter- laboratory study in Canada. J Environ Sci. (2021) 107:218–29. 10.1016/j.jes.2021.01.029 34412784 PMC7929783

[41] Liu TB Chen XY Miao GD. Recommendations on diagnostic criteria and prevention of SARS-related mental disorders. J Clin Psychol Med. (2003) 13:188–191.

[42] Maunder R Hunter J Vincent L Bennett J Peladeau N Leszcz M. The immediate psychological and occupational impact of the 2003 SARS outbreak in a teaching hospital. CMAJ. (2003) 168:1245–51. Available online at: https://www.cmaj.ca/content/168/10/1245.short 12743065 PMC154178

[43] Rogers JP Chesney E Oliver D Pollak TA McGuire P Fusar-Poli P. Psychiatric and neuropsychiatric presentations associated with severe coronavirus infections: a systematic review and meta-analysis with comparison to the COVID-19 pandemic. Lancet Psychiatry. (2020) 7:611–27. 10.1016/S2215-0366(20)30203-0 32437679 PMC7234781

[44] Garcia-Salido A Anton J Martinez-Pajares JD Giralt Garcia G Gomez Cortes B Tagarro A. [Spanish consensus document on diagnosis, stabilization, and treatment of pediatric multisystem inflammatory syndrome related to SARS-CoV-2 (SIM-PedS)]. An Pediatr. (2021) 94:116. 10.1016/j.anpede.2020.09.005 33469560 PMC7808726

[45] Ahmed MU Hanif M Ali MJ Haider MA Kherani D Memon GM. Neurological manifestations of COVID-19 (SARS- CoV-2): a review. Front Neurol. (2020) 11:518. 10.3389/fneur.2020.00518 32574248 PMC7257377

[46] Mao L Jin H Wang M Hu Y Chen S He Q. Neurologic manifestations of hospitalized patients with coronavirus disease 2019 in Wuhan, China. JAMA Neurol. (2020) 77:683–90. 10.1001/jamaneurol.2020.1127 32275288 PMC7149362

[47] Varatharaj A Thomas N Ellul MA Davies NWS Pollak TA Tenorio EL. Neurological and neuropsychiatric complications of COVID-19 in 153 patients: a UK-wide surveillance study. Lancet Psychiatry. (2020) 7:875–82. 10.1016/S2215-0366(20)30287-X 32593341 PMC7316461

[48] Shultz JM Baingana F Neria Y. The 2014 Ebola outbreak and mental health: current status and recommended response. JAMA. (2015) 313:567–8. 10.1001/jama.2014.17934 25532102

[49] Kamara S Walder A Duncan J Kabbedijk A Hughes P Muana A. Mental health care during the Ebola virus disease outbreak in Sierra Leone. Bull World Health Organ. (2017) 95:842–7. 10.2471/BLT.16.190470 29200525 PMC5710077

[50] Cendejas-Bueno E Romero-Gomez MP Escosa-Garcia L Jimenez-Rodriguez S Mingorance J Garcia-Rodriguez J. Lower nasopharyngeal viral loads in the pediatric population. The missing piece to understanding SARS-CoV-2 infection in children? J Infect. (2021) 83: e18–e19. 10.1016/j.jinf.2021.06.009 34133963 PMC8198541

[51] Lee AM Wong JG McAlonan GM Cheung V Cheung C Sham PC. Stress and psychological distress among SARS survivors 1 year after the outbreak. Can J Psychiatry. (2007) 52:233–40. 10.1177/070674370705200405 17500304

[52] Kapfhammer HP Rothenhausler HB Krauseneck T Stoll C Schelling G. Posttraumatic stress disorder and health-related quality of life in long-term survivors of acute respiratory distress syndrome. Am J Psychiatry. (2004) 161:45–52. 10.1176/appi.ajp.161.1.45 14702249

[53] Hawryluck L Gold WL Robinson S Pogorski S Galea S Styra R. SARS control and psychological effects of quarantine, Toronto, Canada. Emerg Infect Dis. (2004) 10:1206–12. 10.3201/eid1007.030703 15324539 PMC3323345

[54] Gerst-Emerson K Jayawardhana J. Loneliness as a public health issue: the impact of loneliness on health care utilization among older adults. Am J Public Health. (2015) 105:1013–9. 10.2105/AJPH.2014.302427 25790413 PMC4386514

[55] Lin CC Chen TY Cheng PY Liu YP. Early life social experience affects adulthood fear extinction deficit and associated dopamine profile abnormalities in a rat model of PTSD. Prog Neuropsychopharmacol Biol Psychiatry. (2020) 101:109914. 10.1016/j.pnpbp.2020.109914 32165120

[56] Neria Y Nandi A Galea S. Post-traumatic stress disorder following disasters: a systematic review. Psychol Med. (2008) 38:467–80. 10.1017/S0033291707001353 17803838 PMC4877688

[57] Santini ZI Jose PE York Cornwell E Koyanagi A Nielsen L Hinrichsen C. Social disconnectedness, perceived isolation, and symptoms of depression and anxiety among older Americans (NSHAP): a longitudinal mediation analysis. Lancet Public Health. (2020) 5:e62–70. 10.1016/S2468-2667(19)30230-0 31910981

[58] Burns A Leavey G Ward M O'Sullivan R. The impact of loneliness on healthcare use in older people: evidence from a nationally representative cohort. J Public Health. (2020) 29:1–10. 10.1007/s10389-020-01338-4 34026705 PMC8131653

[59] Haderlein TP Wong MS Yuan A Llorente MD Washington DL. Association of PTSD with COVID-19 testing and infection in the Veterans Health Administration. J Psychiatr Res. (2020) 144:1–4. 10.1016/j.jpsychires.2020.11.033 33261820 PMC7682935

[60] Qiu J Shen B Zhao M Wang Z Xie B Xu Y. A nationwide survey of psychological distress among Chinese people in the COVID-19 epidemic: implications and policy recommendations. Gen Psychiatr. (2020) 33:e100213. 10.1136/gpsych-2020-100213 32215365 PMC7061893

[61] Lei L Huang X Zhang S Yang J Yang L Xu M. Comparison of prevalence and associated factors of anxiety and depression among people affected by versus people unaffected by quarantine during the COVID-19 epidemic in Southwestern China. Med Sci Monit. (2020) 26:e924609. 10.12659/MSM.924609 32335579 PMC7199435

[62] McGinty EE Presskreischer R Han H Barry CL. Psychological distress and loneliness reported by US adults in 2018 and April (2020). JAMA. (2020) 324:93–94. 10.1001/jama.2020.9740 32492088 PMC7270868

[63] Steinman MA Perry L Perissinotto CM. Meeting the care needs of older adults isolated at home during the COVID-19 pandemic. JAMA Internal Med. (2020) 180:819–20. 10.1001/jamainternmed.2020.1661 32297903

[64] Daly M Robinson E. Anxiety reported by US adults in 2019 and during the 2020 COVID- 19 pandemic: population-based evidence from two nationally representative samples. J Affect Disord. (2021) 286:296–300. 10.1016/j.jad.2021.02.054 33756307 PMC9754788

[65] Xiang Y-T Yang Y Li W Zhang L Zhang Q Cheung T. Timely mental health care for the 2019 novel coronavirus outbreak is urgently needed. Lancet Psychiatry. (2020) 7:228–9. 10.1016/S2215-0366(20)30046-8 32032543 PMC7128153

[66] Cenat JM Mukunzi JN Noorishad PG Rousseau C Derivois D Bukaka J. A systematic review of mental health programs among populations affected by the Ebola virus disease. J Psychosom Res. (2020) 131:109966. 10.1016/j.jpsychores.2020.109966 32087433

[67] Su Z McDonnell D Abbas J Shi L Cai Y Yang L. Secondhand smoke exposure of expectant mothers in China: factoring in the role of culture in data collection. JMIR Cancer. (2021) 7:e24984. 10.2196/24984 34617907 PMC8532012

[68] Callaway E. Delta coronavirus variant: scientists brace for impact. Nature. (2021) 595:17–8. 10.1038/d41586-021-01696-3 34158664

[69] Di Giacomo S Mercatelli D Rakhimov A Giorgi FM. Preliminary report on severe acute respiratory syndrome coronavirus 2 (SARS-CoV-2) Spike mutation T478K. J Med Virol. (2021) 93:5638–43. 10.1002/jmv.27062 33951211 PMC8242375

[70] Burki TK. Lifting of COVID-19 restrictions in the UK and the Delta variant. Lancet Respir Med. (2021) 9:e85. 10.1016/S2213-2600(21)00328-3 34265238 PMC8275031

[71] Moona AA Daria S Asaduzzaman M Islam MR. Bangladesh reported delta variant of coronavirus among its citizen: actionable items to tackle the potential massive third wave. Infect Prevent Pract. (2021) 3:100159. 10.1016/j.infpip.2021.100159 34316588 PMC8239335

[72] Shafi M Liu J Ren W. Impact of COVID-19 pandemic on micro, small, and medium- sized enterprises operating in Pakistan. Res Global. (2020) 2:100018. 10.1016/j.resglo.2020.100018

[73] Abbas J Raza S Nurunnabi M Minai MS Bano S. The impact of entrepreneurial business networks on firms' performance through a mediating role of dynamic capabilities. Sustainability. (2019) 11:3006. 10.3390/su11113006

[74] Local Burden of Disease HIVC. Mapping subnational HIV mortality in six Latin American countries with incomplete vital registration systems. BMC Med. (2021) 19:4. 10.1186/s12916-020-01876-4 33413343 PMC7791645

[75] Paulson KR Kamath AM Alam T Bienhoff K Abady GG Abbas J. Global, regional, and national progress towards sustainable development goal 3.2 for neonatal and child health: all-cause and cause- specific mortality findings from the Global Burden of Disease Study 2019. Lancet. (2021) 398:870–905. 10.1016/s0140-6736(21)01207-1 34416195 PMC8429803

[76] Aqeel M Abbas J Shuja KH Rehna T Ziapour A Yousaf I. The influence of illness perception, anxiety and depression disorders on students mental health during COVID-19 outbreak in Pakistan: a Web-based cross-sectional survey. Int J Hum Rights Healthcare. (2021) 14:1–14. 10.1108/IJHRH-10-2020-0095

[77] Soroush A Ziapour A Abbas J Jahanbin I Andayeshgar B Moradi F. Effects of group logotherapy training on self- esteem, communication skills, and impact of event scale-revised (IES-R) in older adults. Ageing Int. (2021) 46:1–14. 10.1007/s12126-021-09458-2

[78] Aman J Abbas J Lela U Shi G. Religious affiliation, daily spirituals, and private religious factors promote marital commitment among married couples: does religiosity help people amid the COVID-19 crisis? Front Psychol. (2021) 12:657400. 10.3389/fpsyg.2021.657400 34421712 PMC8377757

[79] Azadi NA Ziapour A Lebni JY Irandoost SF Abbas J Chaboksavar F. The effect of education based on health belief model on promoting preventive behaviors of hypertensive disease in the staff of the Iran University of Medical Sciences. Arch Public Health. (2021) 79:69. 10.1186/s13690-021-00594-4 33952339 PMC8097917

[80] Azizi MR Atlasi R Ziapour A Abbas J Naemi R. Innovative human resource management strategies during the COVID-19 pandemic: a systematic narrative review approach. Heliyon. (2021) 7:e07233. 10.1016/j.heliyon.2021.e07233 34124399 PMC8183111

[81] Taufik ER Raza S Zahid H Abbas J Mohd Sobri FA Sidiki SN. Nexus between integrating technology readiness 2.0 index and students' e-library services adoption amid the COVID-19 challenges: implications based on the theory of planned behavior. J Educ Health Promot. (2021) 10. 35372596 10.4103/jehp.jehp_508_21 PMC8974977

[82] Agius S Grech A Grech V. The way in which COVID-19 changed behavior on social media in Malta. Early Hum Dev. (2020) 2020:105255. 10.1016/j.earlhumdev.2020.105255 33248795 PMC7834354

[83] Zhao J Han H Zhong B Xie W Chen Y Zhi M. Health information on social media helps mitigate Crohn's disease symptoms and improves patients' clinical course. Comput Hum Behav. (2021) 115:106588. 10.1016/j.chb.2020.106588

[84] Abbas J Mubeen R Iorember PT Raza S Mamirkulova G. Exploring the impact of COVID-19 on tourism: transformational potential and implications for a sustainable recovery of the travel and leisure industry. Curr Res Behav Sci. (2021) 2:100033. 10.1016/j.crbeha.2021.100033 PMC8690843 38620720

[85] Khazaie H Lebni JY Abbas J Mahaki B Chaboksavar F Kianipour N. Internet addiction status and related factors among medical students: a cross-sectional study in Western Iran. Int Q Commun Health Educ. (2021) 42(2). 10.1177/0272684X211025438 34128427

[86] Lebni JY Toghroli R Abbas J NeJhaddadgar N Salahshoor MR Mansourian M. A study of internet addiction and its effects on mental health: a study based on Iranian University Students. J Educ Health Promot. (2020) 9:205. 10.4103/jehp.jehp_148_20 33062738 PMC7530416

[87] Tang Z Miller AS Zhou Z Warkentin M. Does government social media promote users' information security behavior towards COVID-19 scams? Cultivation effects and protective motivations. Govern Inform Q. (2021) 2021:101572. 10.1016/j.giq.2021.101572 PMC9188430 35719729

[88] Gever VC Talabi FO Adelabu O Sanusi BO Talabi JM. Modeling predictors of COVID- 19 health behavior adoption, sustenance, and discontinuation among social media users in Nigeria. Telemat Informat. (2021) 2021:101584. 10.1016/j.tele.2021.101584 PMC9758446 36569993

[89] Su Z McDonnell D Wen J Kozak M Abbas J Segalo S. Mental health consequences of COVID-19 media coverage: the need for effective crisis communication practices. Global Health. (2021) 17:4. 10.1186/s12992-020-00654-4 33402169 PMC7784222

[90] Abbas J Wang D Su Z Ziapour A. The role of social media in the advent of COVID- 19 pandemic: crisis management, mental health challenges, and implications. Risk Manage Healthcare Policy. (2021) 14:1917–32. 10.2147/RMHP.S284313 34012304 PMC8126999

[91] Carpenter CJ. A meta-analysis of the effectiveness of health belief model variables in predicting behavior. Health Commun. (2010) 25:661–9. 10.1080/10410236.2010.521906 21153982

[92] Jones CJ Smith H Llewellyn C. Evaluating the effectiveness of health belief model interventions in improving adherence: a systematic review. Health Psychol Rev. (2014) 8:253–69. 10.1080/17437199.2013.802623 25053213

[93] Castonguay J Filer CR Pitts MJ. Seeking help for depression: applying the health belief model to illness narratives. Southern Commun J. (2016) 81:289–303. 10.1080/1041794X.2016.1165729

[94] Moradi F Tourani S Ziapour A Abbas J Hematti M Moghadam EJ. Emotional intelligence and quality of life in elderly diabetic patients. Int Q Community Health Educ. (2020). 10.21203/rs.2.18394/v1 33086936

[95] Moradi F Toghroli R Abbas J Ziapour A Lebni JY Aghili A. Hospital managers' skills required and onward challenges: a qualitative study. J Educ Health Promot. (2020) 9:228. 10.4103/jehp.jehp_171_20 33209920 PMC7652088

[96] Abbas J Aman J Nurunnabi M Bano S. The impact of social media on learning behavior for sustainable education: evidence of students from selected universities in Pakistan. Sustainability. (2019) 11:1683. 10.3390/su11061683

[97] Merchant RM Lurie N. Social media and emergency preparedness in response to novel coronavirus. JAMA. (2020) 323:2011–2. 10.1001/jama.2020.4469 32202611

[98] Cerra V Saxena SC. Growth dynamics: the myth of economic recovery. Am Econ Rev. (2008) 98:439–57. 10.1257/aer.98.1.439

[99] Griffith SM Huang W-S Lin C-C Chen Y-C Chang K-E Lin T-H. Long-range air pollution transport in East Asia during the first week of the COVID-19 lockdown in China. Sci Tot Environ. (2020) 741:140214. 10.1016/j.scitotenv.2020.140214 32599400 PMC7295523

[100] Goshit GG Jelilov G Iorember PT Celik B Davd-Wayas OM. Asymmetric effects of monetary policy shocks on output growth in Nigeria: Evidence from nonlinearARDLandHatemi-Jcausality tests. J Public Affairs. (2020) 2020:e2449. 10.1002/pa.244925855820

[101] Jelilov G Iorember PT Usman O Yua PM. Testing the nexus between stock market returns and inflation in Nigeria: does the effect of COVID-19 pandemic matter? J Public Aff. (2020) 20:e2289. 10.1002/pa.2289 32837326 PMC7435360

[102] Iorember PT Jelilov G Usman O Isik A Celik B. The influence of renewable energy use, human capital, and trade on environmental quality in South Africa: multiple structural breaks cointegration approach. Environ Sci Pollut Res Int. (2021) 28:13162–74. 10.1007/s11356-020-11370-2 33179189

[103] Iorember PT Jelilov G. Computable general equilibrium analysis of increase in government agricultural expenditure on household welfare in Nigeria. Afr Dev Rev. (2018) 30:362–71. 10.1111/1467-8268.12344

[104] Iorember PT Goshit GG Dabwor DT. Testing the nexus between renewable energy consumption and environmental quality in Nigeria: the role of broad-based financial development. Afr Dev Rev. (2020) 32:163–75. 10.1111/1467-8268.12425

[105] Goshit GG Iorember PT. Measuring the asymmetric pass-through of monetary policy rate to unemployment in Nigeria: evidence from nonlinear ARDL. Nigerian J Econ Soc Stud. (2020) 62:369–87.

[106] Philip A Iorember PT. Macroeconomic and household welfare impact of an increase in the minimum wage in Nigeria: a computable general equilibrium model. Am J Econ. (2017) 7:249–58. 10.5923/j.economics.20170705.0622499009

[107] Usman O Olanipekun IO Iorember PT Abu-Goodman M. Modelling environmental degradation in South Africa: the effects of energy consumption, democracy, and globalization using innovation accounting tests. Environ Sci Pollut Res Int. (2020) 27:8334–49. 10.1007/s11356-019-06687-6 31900782

[108] Usman O Iorember PT Olanipekun IO. Revisiting the environmental Kuznets curve (EKC) hypothesis in India: the effects of energy consumption and democracy. Environ Sci Pollut Res Int. (2019) 26:13390–400. 10.1007/s11356-019-04696-z 30905016

[109] Wang Q Su M. A preliminary assessment of the impact of COVID-19 on environment - A case study of China. Sci Total Environ. (2020) 728:138915. 10.1016/j.scitotenv.2020.138915 32348946 PMC7195154

[110] Pereira V Temouri Y Patnaik S Mellahi K. Managing and preparing for emerging infectious diseases: avoiding a catastrophe. Acad Manage Perspect. (2020) 34:480–92. 10.5465/amp.2019.0023

[111] Markel H Lipman HB Navarro JA Sloan A Michalsen JR Stern AM. Nonpharmaceutical interventions implemented by US cities during the 1918-1919 influenza pandemic. JAMA. (2007) 298:644–54. 10.1001/jama.298.6.644 17684187

[112] Shuja KH Aqeel M Jaffar A Ahmed A. COVID-19 pandemic and impending global mental health implications. Psychiatria Danubina. (2020) 32:32–5. 10.24869/psyd.2020.32 32303027

[113] Abbas J. The impact of coronavirus (Sars-cov2) epidemic on individuals' mental health: the protective measures of Pakistan in managing and sustaining transmissible disease. Psychiatr Danub. (2020) 32:472–7. 10.24869/psyd.2020.472 33370755

[114] Su Z Wen J Abbas J McDonnell D Cheshmehzangi A Li X. A race for a better understanding of COVID-19 vaccine non- adopters. Brain Behav Immun Health. (2020) 9:100159. 10.1016/j.bbih.2020.100159 33052327 PMC7544597

[115] Yoosefi Lebni J Abbas J Moradi F Salahshoor MR Chaboksavar F Irandoost SF. How the COVID-19 pandemic effected economic, social, political, and cultural factors: a lesson from Iran. Int J Soc Psychiatry. (2020) 67:298–300. 10.1177/0020764020939984 32615838 PMC8107447

[116] Fattahi E Solhi M Abbas J Kasmaei P Rastaghi S Pouresmaeil M. Prioritization of needs among students of University of Medical Sciences: a needs assessment. J Educ Health Promot. (2020) 9:57. 10.4103/0445-7706.281641 32489992 PMC7255571

[117] Pouresmaeil M Abbas J Solhi M Ziapour A Fattahi E. Prioritizing health promotion lifestyle domains in students of Qazvin University of Medical Sciences from the students and professors' perspective. J Educ Health Promot. (2019) 8:228. 10.4103/jehp.jehp_250_19 31867392 PMC6905280

[118] Abbas J. Crisis management, transnational healthcare challenges, and opportunities: the intersection of COVID-19 pandemic and global mental health. Res Global. (2021) 2021:100037. 10.1016/j.resglo.2021.100037

[119] Shoib S Buitrago JETG Shuja KH Aqeel M Filippis R de Abbas J. Suicidal behavior sociocultural factors in developing countries during COVID-19. Encephale (2021) S0013-7006:00183-4. 10.1016/j.encep.2021.06.011 34654566 PMC8457957

[120] Mohammadi A Pishgar E Firouraghi N Bagheri N Shamsoddini A Abbas J. A geodatabase of blood pressure level and the associated factors including lifestyle, nutritional, air pollution, and urban greenspace. BMC Res Notes 14, 416. 10.1186/s13104-021-05830-2 34794504 PMC8600347

[121] Li J., Tourists' health risk threats amid COVID-19 era: Role of technology innovation, Transformation, and recovery implications for sustainable tourism. Front. Psychol. (2021) 12:7619. 10.3389/fpsyg.2021.769175 PMC9022775 35465147

[122] Su Z McDonnell D Li X Bennett B Segalo S Abbas J. COVID-19 Vaccine Donations-Vaccine Empathy or Vaccine Diplomacy? A Narrative Literature Review. Vaccines (Basel). 15:1024. 10.3390/vaccines9091024 34579261 PMC8470866

[123] Toqeer S Aqeel M Shuja KH Bibi A Abbas J. Attachment styles, facebook addiction, dissociation and alexithymia in university students; a mediational model. Nature-Nurture Journal of Psychology. (2021) 1:28–37.

